# Prevalence and Molecular Characterization of *Cyclospora cayetanensis*, Henan, China

**DOI:** 10.3201/eid1710.101296

**Published:** 2011-10

**Authors:** Yang Zhou, Biao Lv, Qiang Wang, Rongjun Wang, Fuchun Jian, Longxian Zhang, Changshen Ning, Kanda Fu, Yaqiang Wang, Meng Qi, Huixia Yao, Jinfeng Zhao, Xiaoshan Zhang, Yanru Sun, Ke Shi, Michael J. Arrowood, Lihua Xiao

**Affiliations:** College of Animal Science and Veterinary Medicine-Henan Agricultural University, Zhengzhou, People’s Republic of China (Y. Zhou, B. Lv, Q. Wang, R. Wang, F. Jian, L. Zhang, C. Ning, M. Qi, H. Yao, J. Zhao, X. Zhang, Y. Sun, K. Shi);; Henan University Huaihe Hospital, Kaifeng, People’s Republic of China (K. Fu, Y. Wang);; Centers for Disease Control and Prevention, Atlanta, Georgia, USA (M.J. Arrowood, L. Xiao)

**Keywords:** protozoa, foodborne infections, enteric infections, prevalence, Cyclospora cayetanensis, modified acid fast stain, PCR, molecular, 18S rRNA, China, dispatch

## Abstract

To determine prevalence of *Cyclospora cayetanensis* infection in Henan, China, we conducted a study of 11,554 hospital patients. Prevalence was 0.70% (95% confidence interval 0.70% ± 0.15%), with all age groups infected. Most cases were found in the summer. Minor sequence polymorphisms were observed in the 18S rRNA gene of 35 isolates characterized.

*Cyclospora cayetanensis*, a protozoan that causes watery diarrhea, fatigue, abdominal pain, weight loss, and inappetence, is endemic to some nonindustrialized countries (*1**–**4*). In industrialized countries, the infection has been traditionally associated with diarrheal illness in travelers to disease-endemic regions. However, since the 1990s, many foodborne and several waterborne outbreaks have been reported in North America (*2**,**3*).

Henan is an agricultural province in central China with a population of >100 million. To better understand the prevalence of cyclosporiasis and genetically characterize *C. cayetanensis*, we conducted a 23-month investigation of cyclosporiasis in patients treated at hospitals in the province.

## The Study

The study was conducted in 2 urban areas, Zhengzhou and Kaifeng. A total of 11,554 (6,939 male; 4,615 female) child and adult patients at 3 hospitals (Huai River Hospital and 155th Liberation Army Hospital, Kaifeng, and Number One People’s Hospital, Zhengzhou) were enrolled in this study during June 2007–December 2008 and July–October 2009. Only data concerning age, sex, and diarrhea presence or absence were made available to laboratorians. One stool specimen from each patient was examined for *Cyclospora* spp. by microscopy of fecal materials that were concentrated by the formalin-ethyl acetate sedimentation method and stained with the modified acid-fast staining technique (*1*). We used the χ^2^ test to compare the frequency of *Cyclospora* spp. infection among patients according to age group and sex and by season of the year. Differences were considered significant if p<0.05.

**Figure 1:**
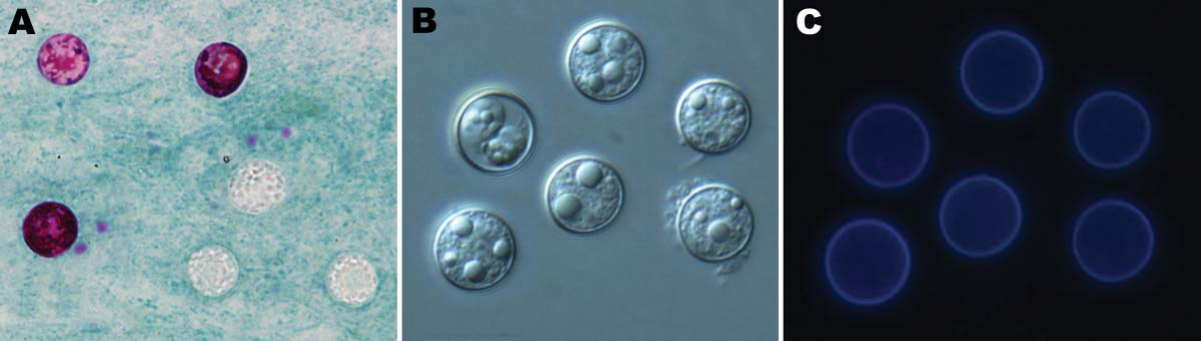
*Cyclospora cayetanensis* oocysts under light microscopy of stool smears stained with the modified acid-fast stain (A), showing differential interference contrast microscopy of wet mount (B), and results of epifluorescence microscopy using a 330–380 nm ultraviolet excitation filter (C). Two oocysts are stained at different intensities (A), and a partially sporulated oocyst is seen (B). Original magnifications ×1,000.

*Cyclospora* oocysts were detected in 81 (0.70%; 95% confidence interval [CI] 0.70% ± 0.15%) of 11,554 patients by microscopy (Table 1). Oocysts were variably stained from light pink to deep purple or remained unstained (Figure 1, panel A). They measured 8.61 ± 0.32 × 8.64 ± 0.33 µm, with a length/width shape index of 1.01 (n = 55; Figure 1, panel B), and showed typical blue autofluorescence under an epifluorescence microscope with a 330–380 nm excitation filter (Figure 1, panel C). Oocysts sporulated at 32°C <13 days in 2.5% potassium dichromate.

**Table 1:** Prevalence and distribution of *Cyclospora cayetanensis* by patient age, sex, and residential area, Henan Province, China, 2009–2010*

Variable	No. infected/ no. patients	Detection rate (95% CI), %
Sex†		
M	47/6,939	0.68 (± 0.01)
F	34/4,615	0.74 (± 0.25)
Age, y		
<6	6/926	0.65 (± 0.52)
7–17	10/678	1.47 (± 0.91)
18–28	13/1,301	1.00 (± 0.17)
29–44	15/2,343	0.64 (± 0.32)
>45	37/6,306	0.59 (± 0.19)
Area†		
Kaifeng	48/6,093	0.79 (± 0.22)
Zhengzhou	33/5,461	0.60 (± 0.20)
Total	81/11,554	0.70 (± 0.15)

*CI, confidence interval. †Pearson correlation >0.05.

*Cyclospora* oocysts were seen in samples from patients in all age groups, although the age group 7–17 years had the highest detection rate (1.47%, 95% CI ± 0.91%; Pearson correlation >0.05) (Table 1). No significant gender difference was found in detection rate; the infection rates for female and male patients were 0.74% (34/4,615, 95% CI ± 0.25%) and 0.68% (47/6,939, 95% CI ± 0.19%), respectively (Pearson correlation >0.05). The overall infection rate of *C. cayetanensis* was similar between Zhengzhou and Kaifeng: 0.60% (95% CI ± 0.19%) versus 0.79% (95% CI ± 0.22%) (Pearson correlation >0.05; Table 1).

**Figure 2:**
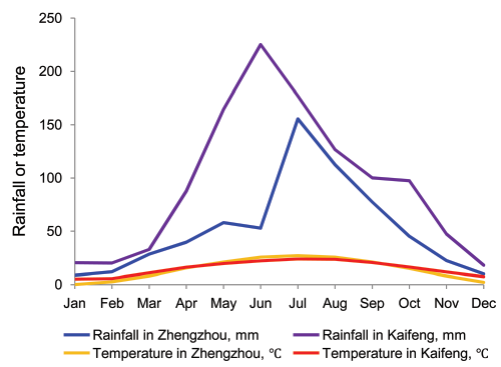
Mean monthly rainfall and mean daily average temperature recorded for Zhengzhou and Kaifeng, Henan Province, China, 1995–2008. Data source: www.chinaweatherguide.com.

The prevalence of cyclosporiasis was markedly seasonal, occurring only during July through November, with a sharp peak in August (Table 2). The occurrence of cyclosporiasis coincided with the rainy season and lagged slightly behind the peaks for mean temperature and precipitation in the year (Figure 2).

**Table 2:** Monthly prevalence of *Cyclospora cayetanensis* in Kaifeng and Zhengzhou, Henan Province, China, 2009–2010*

Month	No. infected/ no. patients	Infection rate (95% CI), %
Jan	0/123	0
Feb	0/89	0
Mar	0/595	0
Apr	0/905	0
May	0/388	0
Jun	0/410	0
Jul†	21/1,1814	1.16 (± 0.49)
Aug‡	44/2,529	1.74 (± 0.51)
Sep†‡	12/1,860	0.65 (± 0.37)
Oct†‡	3/1,638	0.18 (± 0.21)
Nov†‡	1/740	0.14 (± 0.27)
Dec	0/463	0

*CI, confidence interval. †Pearson correlation <0.05. ‡Pearson correlation <0.01.

Among patients in this investigation, 5,533 had records documenting presence or absence of diarrhea at the time of specimen submission. The detection rate of *Cyclospora* oocysts was significantly higher for patients with diarrhea (2.97% or 12/404; 95% CI 2.97 ± 0.52%) than for patients without diarrhea (0.66% or 34/5,129; 95% CI 0.66 ± 0.22%) (Pearson correlation <0.01).

Genomic DNA was extracted from *Cyclospora* oocysts from 35 randomly chosen patients; the oocysts were purified by sucrose gradient centrifugation by using the Mag Extractor-Genome kit (Toyobo Co. Ltd, Osaka, Japan). A nested PCR was used to amplify a 501-bp fragment of the 18S rRNA gene (*5*). All 35 microscopy-positive specimens produced the expected PCR product and were sequenced successfully. The *C. cayetanensis* identity was established by comparing the sequences obtained with a full sequence (AF111183) of the 18S rRNA gene of *C. cayetanensis* from Guatemala and 3 partial sequences (AB368541, AB368542, and AB368543) from Japan. In addition, this comparison revealed the presence of 2 polymorphic sites at nucleotide positions 687 and 696 of the full gene, with a few other inconsistent nucleotide substitutions at other positions. Thus, 3 isolates had a C to T substitution at position 687, and 5 isolates plus AB368542 and AB368543 had a C to T substitution at position 696. Nevertheless, similarities among the 35 *C. cayetanensis* isolates and reference sequences were 99.6%–100% at the 18S rRNA locus. Representative sequences of the partial 18S rRNA gene generated were deposited in the GenBank database under accession nos. GQ292774–GQ292782, FJ009120–FJ009129, and EU860998–EU861002.

## Conclusions

The overall infection rate of 0.70% (95% CI ± 0.15%) in this 23-month investigation in 2 Henan Province cities is similar to data previously obtained in an urban area in the neighboring Anhui Province (0.92%, 95% CI ± 1.04%) (*6*) and studies in Albania (0.63%, 95% CI ± 0.55%) (*7*) and Tanzania (0.91%, 95% CI ± 1.25%) (*8*) but higher than the infection rate in the United Kingdom (0.07%, 95% CI ± 0.07%) (*9*). The rate is significantly lower than those in surveys conducted in healthy populations in other countries (*3**,**10*). The fact that *C. cayetanensis* does not always cause clinical symptoms might have contributed to the differences in infection rates among studies (*3*). In addition, *C. cayetanensis* is mainly spread by consumption of contaminated fresh produce and water (*2**,**3*). In China, persons are less likely to eat raw vegetables and drink unboiled tap water, which are known sources of sporulated *C. cayetanensis* oocysts in nonindustrialized countries (*3*). As expected, in this study the *C. cayetanensis* detection rate was higher for patients with diarrhea than in those without diarrhea because cyclosporiasis has been associated with mild-to-moderate self-limiting diarrhea in children and protracted and severe diarrhea in HIV-positive adults (*2**,**3**,**11*).

In addition to differences in study populations, socioeconomic conditions, and cultural habits, local climatic factors may have contributed to the variation in prevalence of cyclosporiasis observed in different studies. In this study, transmission of *C. cayetanensis* was seasonal; of the 81 cases of cyclosporiasis detected, 95% (95% CI 95% ± 4.7%) occurred during July 1–September 30, the hottest and rainiest months of the year. This result differs from the peak transmission of *C. cayetanensis* in some other geographic areas. For example, in Lima, Peru, *C. cayetanensis* prevalence peaks in warm months (December–May) in the absence of rain (*1*). In contrast, in Haiti where ambient temperature is high year-round, *C. cayetanensis* infection coincides with the cooler part of the year (*10*).

Thus far, 19 species of *Cyclospora* spp. have been described (*3*), but only 4 of them, *C. cayetanensis* and 3 species from nonhuman primates, have been characterized by sequence analysis of the 18S, 5.8S, and 28S rRNA genes and the associated internal transcribed spacer (*3**,**12*). Currently, no reliable genotyping or subtyping tools are available for the investigation of *C. cayetanensis* transmission, the only known *Cyclospora* species that infects humans (*3*). In our study, detection of *C. cayetanensis* in human stool specimens was confirmed by DNA sequencing of the partial 18S rRNA gene. We identified 2 polymorphic sites in the partial 18S rRNA gene of *C. cayetanensis*, although the meaning of the sequence polymorphism remains unclear.

In conclusion, *C. cayetanensis* infects humans in Henan Province at a relatively low frequency but with a marked seasonality. Additional research is needed to determine disease effects, transmission routes, and risk factors for *C. cayetanensis* infection in humans in Henan and elsewhere in China. Research could be facilitated by development of genotyping and subtyping tools for the differentiation and tracking of *C. cayetanensis* isolates.
